# Regeneration of plantlets through somatic embryogenesis from root derived calli of *Hibiscus sabdariffa* L. (Roselle) and assessment of genetic stability by flow cytometry and ISSR analysis

**DOI:** 10.1371/journal.pone.0202324

**Published:** 2018-08-22

**Authors:** Saptarshi Konar, Joydeep Karmakar, Anirban Ray, Sinchan Adhikari, Tapas Kumar Bandyopadhyay

**Affiliations:** 1 Department of Molecular Biology and Biotechnology, University of Kalyani,West Bengal, India; 2 Department of Botany, University of Kalyani, West Bengal, India; College of Agricultural Sciences, UNITED STATES

## Abstract

Induction of somatic embryogenesis and complete plantlet regeneration from callus culture of *Hibiscus sabdariffa* L. var. HS4288 has been made. Leaf and root explants were cultured on Murashige and Skoog (MS) and Driver–Kuniyuki Walnut (DKW) basal media supplemented with different concentrations of synthetic auxins and cytokinins. Root explants on DKW medium supplemented with 2.26μM 2, 4-Dichlorophenoxyacetic acid (2, 4-D) and 4.65μM kinetin (KIN) induced highest percentage (70%) of embryogenic calli. Average number of globular embryos per root derived callus produced within 6 weeks of culture initiation on MS media with different plant growth regulators (PGRs) ranged from 2.27±0.12 to 8.80±0.17 and that of cotyledonary embryos ranged from 0.00 to 2.53±0.20. On DKW medium comparatively more globular embryos (2.70±0.15 to 14.53±0.23) and cotyledonary embryos (0.00 to 8.90±0.17) were produced than that of MS medium. Regeneration of complete plantlets was highest (76.67%) when embryogenic calli with mature somatic embryos were grown on DKW medium containing 2.32μM KIN and 2.22μM 6-Benzyladenine (BA). Plants were primarily hardened in humidity, temperature and light controlled chamber and finally in a greenhouse showed 70% survival ability. Different stages of somatic embryogenesis process in the root derived embryogenic calli were elaborated in detail by morphological, histological and SEM study. The data were statistically analyzed by Duncan Multiple range test (*p* ≤ 0.05) and Principal component analysis (PCA). Flow cytometry and Inter-simple sequence repeats (ISSR) marker analysis confirmed that there was no genetic variation within the regenerated plants.

## Introduction

*Hibiscus sabdariffa* L., an annual plant of family Malvaceae is cultivated in India and many other tropical and subtropical countries. The plant is of great industrial interest for its high quality and comparatively less expensive extraction procedure of bast fiber that is used for making clothes, linen, fishing nets, ropes and allied items. The thick fleshy and cup-shaped calyces of its flowers are used in making jam, liquor and jellies; and also in preparation of Roselle tea [1], [2]. Recently, Roselle fiber has been found to be a suitable natural reinforcement material in composites because of its high-temperature stability and high tensile strength [3]. The plant is also used in several human ailments in folk medicine [4], [5].

Because of its multiple economic utilities, genetic improvement of Roselle warrants deeper research. However, due to the cleistogamous flower of this plant, conventional hybridization is restricted. Moreover, as it is a tetraploid plant (2n = 72) [6], segregation in the generation and purification of the population require a prolonged time in conventional approach of genetic improvement. Attempts however have been made for mutation breeding [7], [8] with meagre success. A tissue culture-independent method using *Agrobacterium* Ti Plasmid reported the production of transgenic Roselle [9].Tissue culture based research with Roselle so far has been insufficient. A few regeneration protocols have been reported but those were mostly through axillary bud culture [10], [11]. Ma’arup et al. [12] established a protocol for diploid and haploid plant regeneration from microspore-derived callus of tetraploid Roselle. Sylvere Sie et al. [13] were the first to report somatic embryogenesis from hypocotyl and cotyledon-derived calli; the root derived calli showed poor response to the induction of somatic embryos [13]. In this background, our objective was to test the possibility of induction of somatic embryogenesis from root and leaf derived calli of *H*. *sabdariffa* L. var. HS4288 that were not tested before. We also studied the different stages of somatic embryo development, embryo maturation and their differentiation into complete plants as well as their acclimatization under controlled environment in the lab and finally in the field.

Genetic stability analysis of *H*. *sabdariffa* during somatic embryogenesis may be useful for understanding the genetic basis of variation. Genetic variation may occur at the chromosomal level, such as, changes in chromosome structure, chromosome numbers as in polyploidy and aneuploidy or at the DNA sequence level [14]. To check the genetic fidelity of the regenerants, the nuclear flow cytometry along with ISSR marker analysis have been accomplished in several plants [15], [16]. In the present work, flow cytometry and ISSR analysis system have been attempted to investigate the genetic fidelity of the somatic embryo derived plantlets of *H*. *sabdariffa* L. var. HS4288.

## Materials and methods

### Chemicals

Ultrapure chemicals and solvents were used in the present study. The plant growth regulators (PGRs), L-Glutamine, RNase A and propidium iodide were obtained from Sigma-Aldrich, USA; basal culture medium, agar and sucrose from HiMedia, India; potassium nitrate and mercuric chloride from Merck, India were used throughout the experiments. The molecular biology grade chemical reagents were procured from Sisco Research Laboratory (SRL), India and Sigma-Aldrich, USA. PCR related components from NEB, USA; ISSR primers from Integrated DNA Technologies, USA and 100 bp DNA ladder from GCC Biotech Pvt. Ltd., India were used during the study.

### Source of plant material, sterilization and explant preparation

The seeds of high yielding variety HS 4288 of *H*. *sabdariffa*, were collected from Central Research Institute for Jute and Allied Fibres (CRIJAF), Barrackpore, West Bengal, India. Preliminary trial showed 55–60% or less germination of the seeds. Therefore, the seeds were pre-treated with 2% KNO_3_ (w/v) solution for 24 h in the dark for better germination (more than 75%). Seeds were then sterilized with 0.1% (w/v) mercuric chloride for 5 min followed by repeated washing with sterile distilled water and allowed to germinate aseptically. A healthy plant from the germinating seeds was randomly selected and multiplied by axillary bud culture in the fresh MS basal medium devoid of any PGRs. The leaves and roots of these 45 days old plants from axillary bud culture (termed as mother plant) were excised into small pieces (leaf, 1cm×1cm and root, 1cm) and pricked with sterile scalpel before inoculation in diverse culture media for the induction of embryogenic callus. Routine axillary bud culture on PGRs free medium was also maintained to conserve the mother plants in the laboratory.

### Culture media and condition for induction of callus and somatic embryos

The effects of embryogenic callus formation and induction of somatic embryos was evaluated in two basal media, MS [17] and DKW [18] which were supplemented with 2.26 μM 2,4-D and 2.32–9.30 μM KIN; 2.26 μM 2,4-D and 2.22–8.88 μM BA; 2.68 μM NAA and 2.22–8.88 μM BA. Both the basal media, devoid of any PGRs, were used as control in all experiments. All of the culture media were supplemented with 87.64 μM sucrose, 75 μM glutamine and 0.8% (w/v) agar. The pH of culture media was adjusted to 5.7 before autoclaving at 1.06 kg cm^-2^ pressure and 121°C for 15 min. The cultures were maintained at 25 ± 2°C, either in dark or 16 h photoperiod under 50 μmol m^-2^ s^-1^ cool white fluorescent light (Philips, India) and 60–70% relative humidity.

### Conversion of somatic embryos into plantlets

The root derived calli with highly matured somatic embryos were transferred to another sets of MS and DKW medium containing different combinations of 2.32 μM KIN and 2.22–8.88 μM BA to calculate the conversion frequencies of somatic embryos into plantlets. Initially it was observed that the first cotyledonary leaves appeared at least after 40–45days in MS medium, whereas, in DKW it initiated within a minimum time span of 14 days. Hence, further studies on somatic embryo conversion were carried out using DKW medium with the aforementioned PGRs concentrations.

### Pre-hardening and acclimatization of regenerated plantlets

Newly formed plantlets with small roots were dissected from the callus clump and cultured for one month onto the PGRs free half-strength DKW medium for better rooting. Young plantlets of 3–4 cm in size with 5–6 roots were rinsed carefully and transplanted in the plastic pro-trays containing coco-peat and coarse sand (2:1) mixture for primary hardening. The plants in pro-trays were incubated in a plant growth chamber having 25°C temperature and 75% relative humidity with occasional misting of water and kept for one month with this condition until the new leaves sprouted. One month old primary hardened plants were then transferred in earthen pots containing a mixture of soil, sand and organic manure in 1:1:1 ratio for the purpose of secondary hardening.

### Histology and scanning electron microscopy

The root derived calli with different stages of somatic embryos were fixed for histology and scanning electron microscopy following the method of our previous report on somatic embryogenesis of curry leaf [19]. For histology study, the thin sections (10 μm) were cut using an electronic rotary microtome (MICROMHM 340E, Thermo Fisher Scientific, Germany) followed by staining with Hematoxylin and Eosin stain. The section strips were further washed with xylol, mounted with DPX (BDH) and examined under a compound microscope (Leica, DME, Germany). For SEM study, embryogenic tissues were finally dried in a critical point drier (HCP-2, Hitachi, Japan) and coated with gold sputter prior to the examination under a scanning electron microscope (ZEISS EVO LS 10, Germany) at 10kV with different magnifications.

### Flow cytometry

The nuclear suspensions of the young, fresh root tissues (~50 mg) from the randomly selected plantlets of both mother and somatic embryo derived plants of *H*. *sabdariffa* var. HS 4288 were prepared by chopping with a fine razor blade in 500 μl of the Galbraith’s nuclei isolation buffer [20] and filtered through a double layered 50 μm nylon mesh to remove cell fragments and large debris. The young root tissue of rice plant (*Oryza sativa* ‘IR36’) was treated similarly and used as the reference standard (1.01pg/2C) [21] The nuclei of both the plants were then treated with 50 μg ml^-1^ RNase A and simultaneously stained with 50 μg ml^-1^ of propidium iodide (PI) prior to incubation on ice for 10 min. The prepared samples were analyzed on a flow cytometer (BD Accuri C6). A linear PI fluorescence area (PI-A) vs. PI fluorescence width (PI-W) plot was drawn to eliminate clumps and aggregates using qualitative gating. A PI fluorescence histogram (PI-A) was drawn to view the nuclear DNA content and the positions of the peak on the histograms were compared to determine the ploidy stability between the mother and somatic embryo derived plants. The 2C value of the regenerated plants was estimated using the following formula [22]

$$2C\;value\;of\;regenerated\;plant\;(pg)=\frac{Sample\frac{G0}{G1}peak\;MFI}{Standard\frac{G0}{G1}peak\;MFI}\times 2C\;value\;of\;standard(pg)$$

### Genomic DNA extraction and ISSR marker analysis

Total genomic DNA was extracted using CTAB extraction method [23] from both mother and somatic embryo derived plants of *H*. *sabdariffa* var.HS 4288. Following RNase treatment the quality of extracted DNA was assessed on 0.8% agarose gel stained with ethidium bromide (0.5 μg μl^-1^) and finally the quantification of DNA was done using Nanodrop spectrophotometer (Nanodrop 2000, Thermo Scientific, USA). A bulk DNA sample was prepared separately by pooling an equal amount of DNA from axillary bud derived individuals (mother plants) and used for PCR amplification. All the DNA samples were diluted to 30 ng μl^-1^ before ISSR analysis. A factorial experiment with varying concentrations of MgCl_2_ (1.5, 2.0 and 2.5 mM) and Taq DNA polymerase (0.5, 0.75 and 1 U) was performed in different PCR cycles to optimize the best PCR conditions and to obtain the best amplification results. For initial screening, a total of 25 ISSR primers were selected either from UBC series or from the previously published literatures [24–28]. Out of these twenty five primers, 15 primers (14–18 mer) both 5', 3' anchored and unanchored were finally selected based on their ability of production of reproducible and scorable loci. Optimized PCR reaction mixtures of 25 μl volume comprised of 2.5 μl of 10X PCR buffer, 200 μM dNTP mix, 2 mM MgCl_2_, 10 pmol primer, 1.0 U of Taq polymerase and 30 ng of template DNA. PCR amplifications were carried out in a thermal cycler (Veriti, Applied Biosystems, USA) and cycling condition was programmed for 4 min at 94°C followed by 40 cycles at 94°C for 15 s, 50–60°C for 15 s, 72°C for 1 min 15 s, with a final incubation at 72°C for 7 min. The amplified fragments were analyzed on 1.8% agarose gel containing 0.5 μg μl^-1^ ethidium bromide in the presence of 100 bp DNA ladder and finally visualized using Gel-documentation system (Bio-Rad XRS+, Bio-Rad, USA).

### Experimental design and statistical analyses

The explants forming the embryogenic calli and the frequency of different stages of somatic embryos within the callus were scored under a stereo zoom microscope (SZH, Olympus, Tokyo, Japan) after 4 and 6 weeks of cultures, respectively. The requirement of minimum days needed for germination was calculated based on the appearance of first cotyledonary leaf; and the frequency of germinated plantlets was scored at 45^th^ day from the date of inoculation in fresh germinating media. All of the experiments were carried out in a randomized block design and each set of experiment comprised of ten explants or cultures in triplicate. The results were analyzed as mean ± standard errors (SE) and the percentage data were arcsine-transformed following the method of our previous study [28], [29]. The significant difference (*p* ≤ 0.05) among the mean was assessed by analysis of variance followed by Duncan’s multiple range test (DMRT) using MSTAT-C software (ver. 1.41, Michigan State University). Principal component analysis (PCA), a multivariate statistical technique has been carried out with the findings from Table 1, pertained to the responses of embryogenic callus formation by root and leaf explants. The combination of various growth regulators in both DKW and MS medium were considered to be various treatments presented at the bi-plots, which shows the overall distribution of the considered parameters. The analysis was performed at 95% confidence interval with Origin Pro 9.0 software package to delineate the cumulative distribution of two different explants in relation to considered basal medium fortified with various PGRs. Flow cytometry data were analyzed with BD Accuri C6 software performed independently on three different days with ten samples from each group. For ISSR analysis, the PCR reactions were repeated thrice for each primer to ensure the reproducibility of results and only clear and reproducible bands were scored for data interpretation.

**Table 1:** Effects of two basal media MS and DKW augmented with different combinations and concentrations of plant growth regulators (PGRs) on the induction of embryogenic callus from two different explants (root and leaf) of *H*. *sabdariffa* L. var. HS 4288.

Basal culture medium	Concentration of PGRs (μM)				Percentage of explants forming embryogenic callus	
	2,4-D	KIN	NAA	BA	Root	Leaf
MS	0.0	0.0	0.0	0.00	0.00^m^	0.00^m^
		2.32	-	-	46.67(43.28)^cdef^	26.67(31.31)^hijk^
	2.26	4.65	-	-	50.00(45.00) ^cdef^	30.00(33.21)^ghij^
		9.30	-	-	53.33(46.72)^cde^	36.67(37.46)^fghi^
		-	-	2.22	23.33(28.66)^ijk^	6.67(15.34)^lm^
	2.26	-	-	4.44	36.67(37.46)^fghi^	20.00(26.57)^jkl^
		-	-	8.88	40.00(39.23)^efgh^	26.67(31.31)^hijk^
	-	-		2.22	6.67(15.34)^lm^	0.00^m^
	-	-	2.68	4.44	16.67(24.35)^jkl^	0.00^m^
	-	-		8.88	20.00(26.57)^jkl^	6.67(15.34)^lm^
DKW	0.0	0.0	0.0	0.00	0.00^m^	0.00^m^
		2.32	-	-	60.00(50.77)^abc^	43.33(40.98)^defg^
	2.26	4.65	-	-	70.00(56.79)^a^	53.33(46.72)^cde^
		9.30	-	-	66.67(54.94)^ab^	56.67(49.02)^bcd^
		-	-	2.22	36.67(37.46)^fghi^	16.67(24.35)^jkl^
	2.26	-	-	4.44	46.67(43.28)^cdef^	26.67(31.31)^hijk^
		-	-	8.88	46.67(43.28)^cdef^	30.00(33.21)^ghij^
	-	-		2.22	16.67(24.35)^jkl^	6.67(15.34)^lm^
	-	-	2.68	4.44	30.00(33.21)^ghij^	13.33(21.13)^klm^
	-	-		8.88	26.67(31.31)^hijk^	16.67(24.35)^jkl^

Values are expressed as means of three replicated experiments, each with ten explants per treatment. Means followed by the same letters in superscript within columns are not significantly different at *p ≤0*.*05* level according to Duncan multiple range test (DMRT). Values in the parentheses are the arc sine transformations of the percentage data. All data were taken after 4 weeks of culture.

## Results and discussion

### Embryogenic callus induction

**Fig 1:**
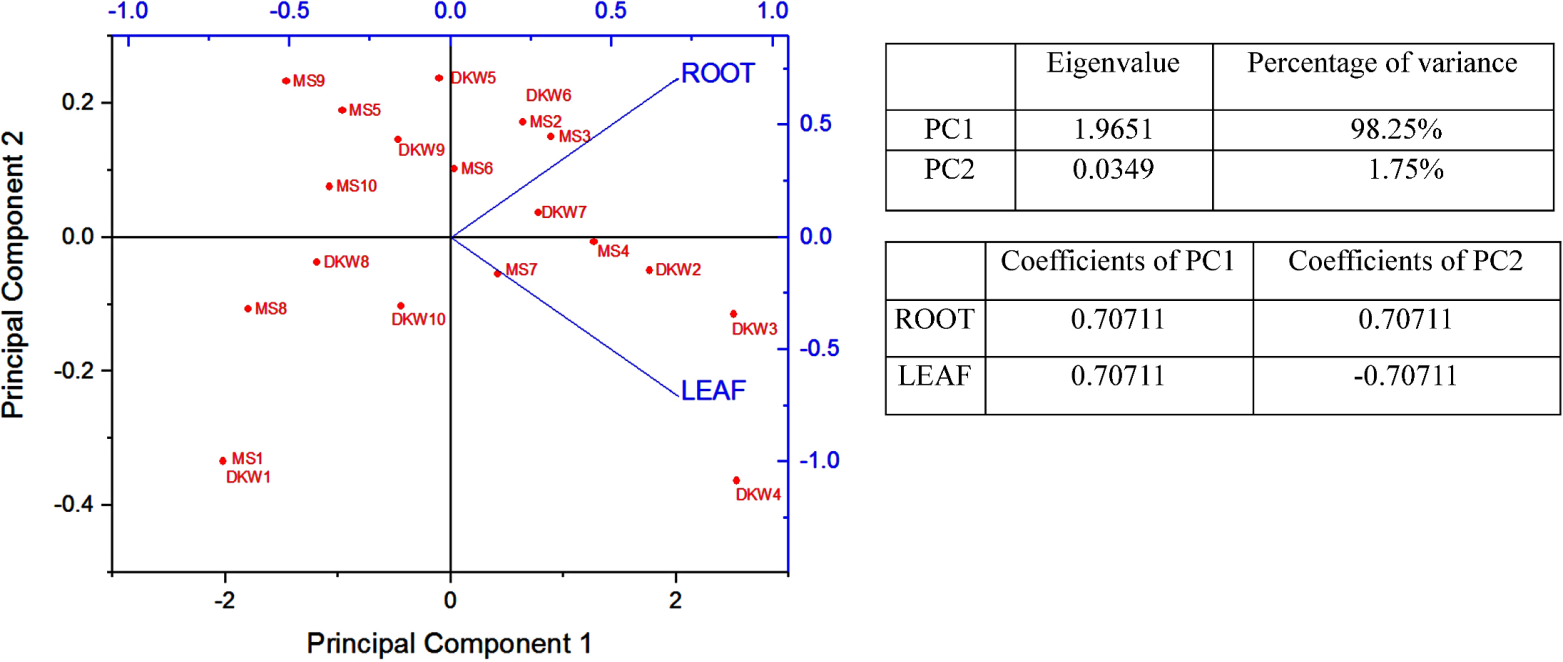
Bi-plot with first and second components of PCA based on the frequency of embryogenic callus induction from root and leaf explants of *H*. *sabdariffa* var. HS 4288, cultured on DKW and MS medium supplemented with different concentrations and combinations of plant growth regulators. Distributional bi-plot with two different medium supplemented with different concentrations and combinations of plant growth regulators (PGRs) shown in the figure as, MS1 or DKW1 = No PGRs; MS2 or DKW2 = 2.26 μM 2,4-D + 2.32 μM KIN; MS3 or DKW3 = 2.26 μM 2,4-D + 4.65 μM KIN; MS4 or DKW4 = 2.26 μM 2,4-D + 9.30 μM KIN; MS5 or DKW5 = 2.26 μM 2,4-D + 2.22 μM BA; MS6 or DKW6 = 2.26 μM 2,4-D + 4.44 μM BA; MS7 or DKW7 = 2.26 μM 2,4-D + 8.88 μM BA; MS8 or DKW8 = 2.68 μM NAA + 2.22 μM BA; MS9 or DKW9 = 2.68 μM NAA+ 4.44 μM BA; MS10 or DKW10 = 2.68 μM NAA+ 8.88 μM BA. The eigenvalues of two PCs and their percentage of variance have been shown in this figure. In this bi-plot, PC1 correlated positively with both root and leaf explants, whereas PC2 positively correlated with the root explant only.

The efficiency of MS and DKW media supplemented with different PGRs on the induction of embryogenic callus from root and leaf explants of the test plant was compared and results are presented in Table 1. It was noted that MS medium supplemented 2.26 μM 2, 4-D and 9.30 μM KIN induced maximum 53.33% of root explant to form embryogenic callus. In case of leaf explant, the same medium gave 36.67% of embryogenic callus. On DKW media supplemented with 2.26 μM 2, 4-D and 4.65 μM KIN, the highest percentage of embryogenic callus formation was 70% from root explant, but in case of leaf explant 2.26 μM 2, 4-D and 9.30 μM KIN yielded highest percentage of embryogenic callus formation (56.67%). Results clearly indicated that DKW medium is more suitable than MS medium when supplemented with 2, 4-D and varying concentrations of KIN (Table 1). Results further indicated that higher percentage of embryogenic callus was produced from root explant than that of leaf explant. Duncan multiple range test (DMRT) using arcsine-transformation of the data of the Percentage value in Table 1 showed that the efficiency of root explants forming embryogenic callus is significantly different (*p* ≤ 0.05) from that of leaf derived callus. Now, to delineate the cumulative distribution of two different types of explant for the induction of embryogenic callus on MS and DKW culture medium a concerted effort has also been made by the use of principal component analysis (PCA). The reliability of this statistical tool has now been proved in different plant tissue culture experiments to conclude their findings [30], [31]. In PCA, the data based on the percentage of root and leaf explants forming embryogenic callus (Table 1), were projected over first two principal components. The eigenvalues of two PCs (PC1: 1.9651, PC2: 0.0349) and their percentage of variance (PC1: 98.25%, PC2: 1.75%) are presented in Fig 1. In this bi-plot, PC1 correlated positively with both root and leaf explants, whereas PC2 positively correlated with the root explant only (Fig 1). Root explants exhibiting positive correlation both in PC1 and PC2 indicated the promising responses accounting to the closer proximity with different media when compared to that of the leaf explants with same treatment. The DKW medium at varying concentrations of growth regulators was closely associated with root and leaf explants, when compared to the MS medium at the respective concentrations of PGRs. It suggests that DKW medium might be considered as a better option than that of MS medium to get the desired responses from the explants of *H*. *sabdariffa* var. HS 4288. In an earlier publication of somatic embryogenesis in two varieties (var. *sabdariffa* and var. *altissima*) of *H*. *sabdariffa* [13], it was reported that the root was not suitable explant for embryogenesis study. Our data from Table 1 and PCA analysis clearly indicated that the root is more suitable explant than the leaf, and DKW is better culture medium than MS for our test plant. The apparent variation in the results might be due to genetic variation between the variety used in the present study and that of the varieties used by the authors in the earlier publication [13].The data with respect to the influence of media, our results support the contention of Sylvere Sie et al. [13] that DKW is better culture medium than MS for induction of somatic embryogenesis in *H*. *sabdariffa*.

**Fig 2:**
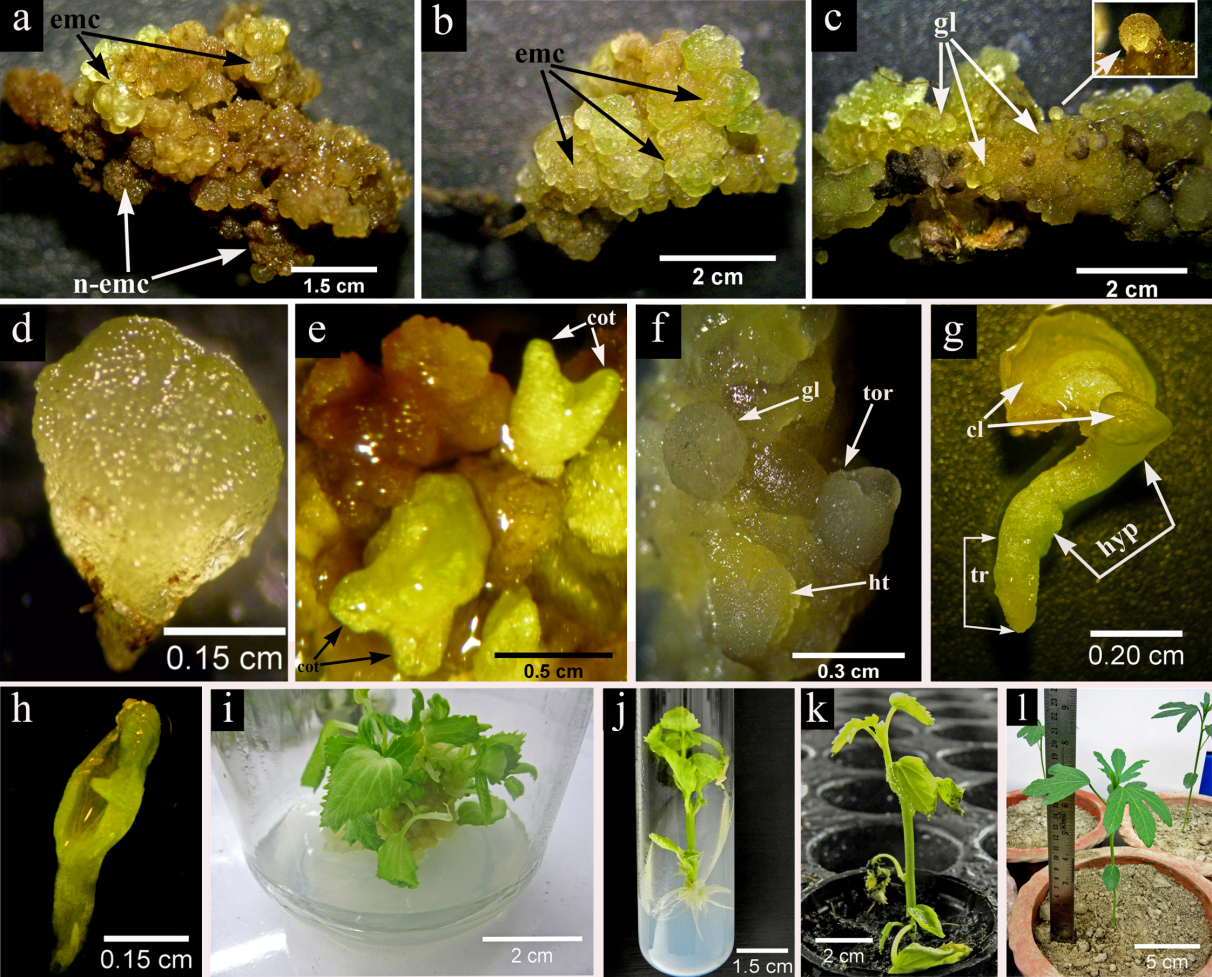
Differential pattern of somatic embryo development in DKW basal medium (2.26 μM 2,4-D + 4.65 μM KIN) and somatic embryo germination, conversion (2.32 μM KIN + 2.22 μM BA) and acclimatization in *H*. *sabdariffa* var. HS 4288. (a) A patch of both light green embryogenic (emc) as well as brown non embryogenic (n-emc) callus developed from the leaf explant after 4 weeks of culture; (b) 4-weeks old root derived green and highly granular embryogenic (emc) callus, (c) characterized by distinct globular embryo (gl) like structures which appeared on the surface of calli either in clump or singly (inset); (d) formation of isolated prominent bipolar heart shaped (ht) embryo with distinct shoot and root pole within 2^nd^ week; (e) asynchronous somatic embryo development such as globular (gl), heart (ht), torpedo (tor) shaped structures on 4^th^ week and (f) final differentiation at cotyledonary stage with clear notch and distinct cotyledons after 6 weeks of second subculture on the aforementioned medium, (g) epigeal pattern of somatic embryo germination characterized by distinct green colored cotyledonary leaves (cl), elongated hypocotyl (hyp) and tap root (tr), (h) abnormal somatic embryo having a single cotyledon (i) formation of clump of plantlets from cotyledonary stage embryos after 45 days in the conversion medium containing DKW basal medium supplemented with 2.32 μM KIN and 2.22 μM BA; (j) 6-weeks old somatic embryo derived healthy rooted plantlets in PGRs free DKW medium; (k) one month old primary hardened plant in the pro-trays containing coco-peat and coarse sand (2:1) mixture; (l) two month old secondary hardened plant in the earthen pot containing a mixture of soil, sand and organic manure (1:1:1).

The morphological study indicated that leaf derived callus was granular in texture with light green color and the embryos grew on their outer surface but with time they slowly turned into brown (Fig 2A) and lost their embryogenic potentiality. In contrast, the root derived callus was prominently embryogenic, highly granular with distinct globular structures (Fig 2B) resembling to typical somatic embryos that appeared on the surface of calli more or less after 4 weeks in culture (Fig 2C) containing 2.26 μM 2, 4-D and 2.32 μM kinetin. The effect of 2, 4-D in presence of other cytokinins on embryogenic callus induction has also been reported in *H*. *sabdariffa* [12], [13] and our results also indicated that 2, 4-D is a positive inducer for somatic embryogenesis in *H*. *sabdariffa* L. var. HS 4288.

### Maturation of somatic embryos derived from root callus

Initially somatic embryogenesis started by the formation of globular embryos on the surface of the root derived callus. For maturation study, the areas of the callus showing globular embryos were aseptically dissected out with a small base of original callus and transferred separately to MS and DKW medium supplemented with the same combination and concentration of PGRs used in the induction for embryogenic callus. After 6 weeks in culture, the average number of globular and cotyledonary somatic embryos were calculated and the results are presented in Table 2. It is apparent that for maturation of somatic embryos on DKW medium supplemented with suitable concentrations of 2, 4-D and KIN yielded higher numbers of globular (ranged from 11.13±0.12 to 14.53±0.23) and cotyledonary (ranged from 6.87±0.20 to 8.90±0.17) somatic embryos. During the time of regeneration the morphological features of different differentiations stages up to plantlet formation were studied. On DKW medium supplemented with 2.26 μM 2, 4-D and 4.65 μM KIN bipolar heart shaped embryo (Fig 2D) appeared within 2^nd^ week of culture. The somatic embryos at this stage were loosely attached with their callus base and could be separated easily by fine needle and forceps. The frequency and asynchronous embryo development with globular, heart and torpedo shaped structure (Fig 2E) was sharply increased in next two weeks. In the 4^th^ week onwards, the torpedo shaped embryo transformed into cotyledonary stage having a distinct notch and well demarcated cotyledons (Fig 2F).

**Table 2:** Influence of various concentrations of plant growth regulators (PGRs) supplemented media on somatic embryos development from root derived embryogenic callus of *H*. *sabdariffa* var. HS 4288.

Basal culture medium	Concentration of PGRs (μM)				Average number of somatic embryos	
	2,4-D	KIN	NAA	BA	Globular	Cotyledonary
	0.0	0.0	0.0	0.00	0.00^n^	0.00^i^
		2.32	-	-	6.83±0.15^f^	2.10±0.12^fg^
	2.26	4.65	-	-	7.53±0.18^e^	2.37±0.32^ef^
		9.30	-	-	8.80±0.17^d^	2.53±0.20^e^
MS		-	-	2.22	2.73±0.12^l^	0.00^i^
	2.26	-	-	4.44	3.23±0.15^k^	0.00^i^
		-	-	8.88	4.03±0.18^ij^	1.37±0.12^h^
	-	-		2.22	2.27±0.12^m^	0.00^i^
	-	-	2.68	4.44	3.20±0.21^k^	0.00^i^
	-	-		8.88	4.03±0.17^ij^	0.00^i^
	0.0	0.0	0.0	0.00	0.00^n^	0.00^i^
		2.32	-	-	11.13±0.12^c^	6.87±0.20^c^
	2.26	4.65	-	-	14.53±0.23^a^	8.90±0.17^a^
		9.30	-	-	13.03±0.19^b^	7.68±0.12^b^
DKW		-	-	2.22	3.17±0.2^k^	0.00^i^
	2.26	-	-	4.44	4.07±0.12^h^	1.87±0.15^g^
		-	-	8.88	6.10±0.17^g^	3.20±0.21^d^
	-	-		2.22	2.70±0.15^lm^	0.00^i^
	-	-	2.68	4.44	3.73±0.12^j^	0.00^i^
	-	-		8.88	4.10±0.12^hi^	1.97±0.12^g^

Values are expressed as mean ± standard error (±SE) of three replicated experiments, each with ten explants per treatment. Means followed by the same letters in superscript within columns are not significantly different at *p ≤ 0*.*05* level according to Duncan multiple range test (DMRT). All data were taken after 6 weeks of culture.

In the last few years, the totipotency of root explants has been established in different plants, as in *Arabidopsis* [32], *Brassica oleracea* [33] *Coriandrum sativum* [34], *Limonium* Misty Blue [28] etc. Practically the plant parts which are used as explants contain a variety of tissues that are ready to express pluripotency or totipotency but they are often inhibited by the neighboring tissues. Such type of explants when cultured *in vitro* could make them free from the inhibitions and express their pluri- or totipotency [35]. Sugimoto et al. [36] established that during callus-mediated regeneration the explants like petals and cotyledons exhibit the root meristem characteristics. In some cases, the rooting-related genes are not expressed properly within the callus; therefore, it does not show any organogenic and embryogenic responses. The use of young root tissue as a source of explant is worth exploring because it has highest rooting related genes expression capacity whereas in callus obtained from other plant parts, it may be difficult to express such genes [35].Our results supported the suitability of the use of young root explants for better induction and maturation of somatic embryos in culture.

### Plantlets formation

The conversion of somatic embryos into plantlets is a crucial step in somatic embryogenesis process. Previously, the somatic embryogenesis was claimed in two varieties of *H*. *sabdariffa* [13] but no plantlet regeneration was documented. In the present protocol, the germination of matured somatic embryo was clearly demonstrated by photomicrographs. In our test plant, the epigeal pattern of somatic embryo conversion appeared to be similar to that of zygotic embryo. The first leaves in the form of cotyledon turn green along with the first emergence of hypocotyl and tap root (Fig 2G). During conversion some of the abnormal somatic embryos with single cotyledon were also appeared (Fig 2H). The DKW basal medium fortified with 2.22 μM BA and 2.32 μM KIN was found to be the most effective, which promoted the emergence of the first cotyledonary leaves within a time span of 14 days and the maximum conversion frequency was 76.67% after 45 days (Table 3). The culture medium devoid of any PGRs produced lowest conversion frequency. In some plants like *Sapindus mukorossi*, the presence of 2,4-D in culture medium triggers somatic embryo induction and maturation but its substitution with cytokinins [37] promoted somatic embryo germination. Similar results have also been found in our study with *H*. *sabdariffa* L. var. HS 4288.

**Table 3:** Effect of different concentrations of cytokinins on somatic embryo conversion into plantlets.

Concentration of cytokinins (μM) in DKW medium		Appearance of the first cotyledonary leaves after days		Percent conversion of somatic embryos into plantlets after 45 days	
KIN	BA				
0.0	0.0		37.67±1.20^a^		26.67(31.31)^d^
	2.22		14.33±0.67^e^		76.67(61.34)^a^
2.32	4.44		20.67±0.88^d^		63.33(52.54)^ab^
	6.66		25.67±0.33^c^		53.33(46.72)^bc^
	8.88		30.00±1.15^b^		43.33(40.98)^c^

Values are expressed as mean or mean ± standard error (±SE) of three replicated experiments. Means followed by the same letters in superscript within columns are not significantly different at *p ≤0*.*05* level according to Duncan multiple range test (DMRT). Values in the parentheses are the arc sine transformations of the percentage data.

### Rooting, ex-vitro acclimatization and field transfer

The somatic embryo derived small plantlets with primary root system were separated from the clump of plantlets (Fig 2I) after 45 days and transferred onto the PGRs free DKW medium for better growth. The six weeks old healthy rooted plants (Fig 2J) were then transferred for hardening. The rooted plantlet (3–4 cm) having 5–6 leaves were transplanted in the pro-trays containing coco-peat and coarse sand (2:1) mixture for primary hardening (Fig 2K)The suitable use of coco-peat and coarse sand mixture during hardening has also reported by Bhattacharya et al. [38] in *Anthurium andraeanum* and Bose et al. [28] in *Limonium* hybrid ‘Misty Blue’. In the second phase of hardening, the earthen pot filled with a mixture of soil, sand and organic manure (1:1:1) was found to be most appropriate with 70% survival ability and healthy growth (Fig 2L). Morphologically the regenerated plants showed similarity with that of the mother plant.

### Histology and scanning electron microscopy

**Fig 3:**
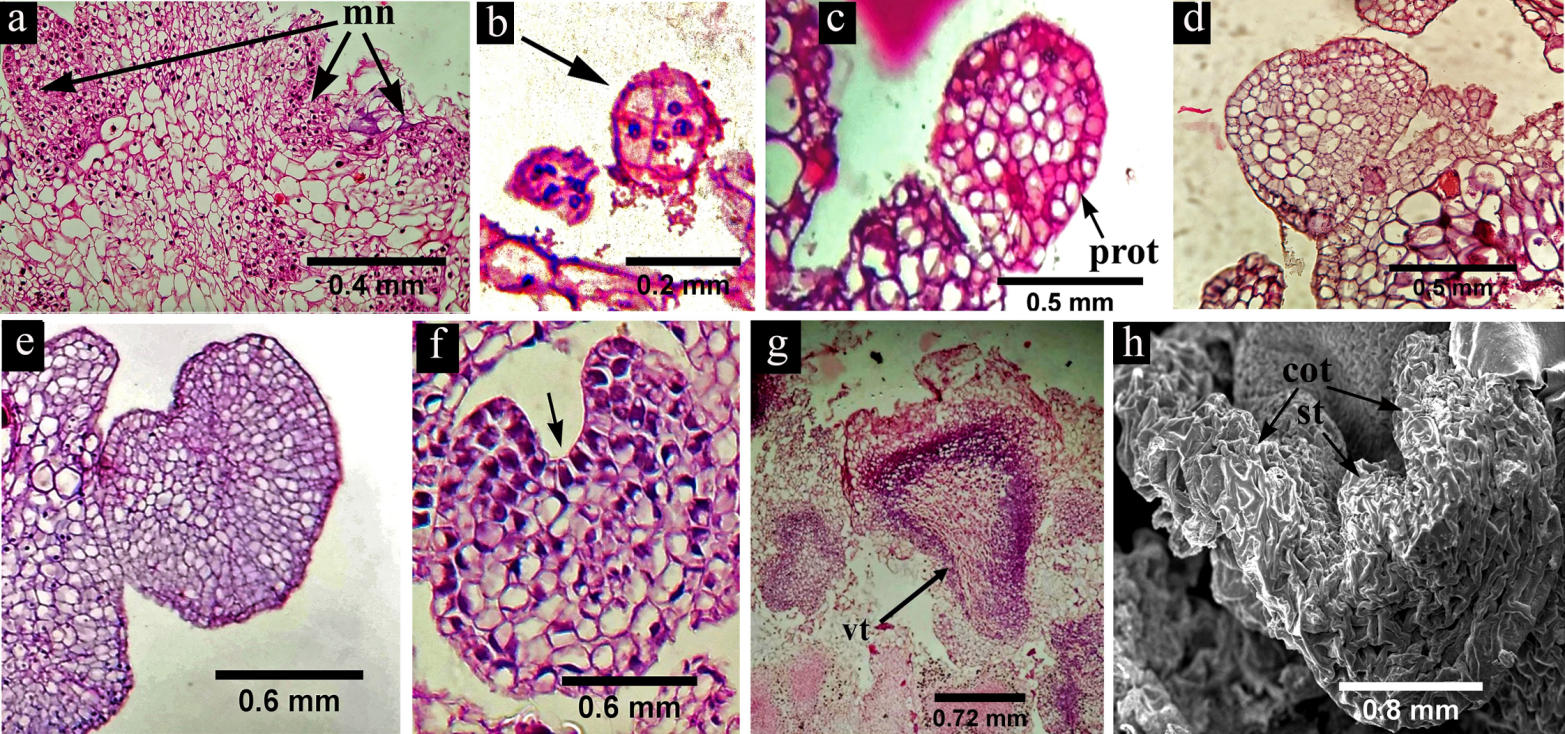
Histology and scanning electron microscopic view of somatic embryo development in *H*. *sabdariffa* var. HS 4288. (a) The cross section of the embryogenic callus showing some meristematic nodules (mn) with darkly stained cells and prominent nuclei; (b) 8-celled pro-embryo (arrow mark) representing the first stage of somatic embryo initiation; (c) globular embryo with clear protoderm (prot); (d) early heart shape, (e) heart shape and (f) late heart shape somatic embryo with incipient cotyledon and clear cotyledonary notch (arrow mark); (g) torpedo shaped embryo with well demarcated vascular trace (vt); (h) germinating embryo with two cotyledons (cot) and elongated shoot tip (st).

The developmental pattern of somatic embryos of *H*. *sabdariffa* was examined by histo-differentiation and scanning electron microscopy. The cross section of the embryogenic callus showed a few meristematic nodules (Fig 3A) on their surface which are characterized by darkly stained cells with prominent nucleus. The somatic embryos originated from the surface of these nodules as an 8-celled pro-embryo (Fig 3B) which was probably the first stage of somatic embryo initiation. These pro-embryos further differentiated into well-developed globular embryo with clear protoderm (Fig 3C). In the next stage of development the embryos were characterized by early heart (Fig 3D), heart (Fig 3E) and late heart (Fig 3F) appearance with clear cotyledonary notch. Heart shape and bipolar differentiation of the globular embryo indicated their independent nature of growth and development like that of zygotic embryos. In late developmental stage the embryos were further elongated and transformed into torpedo shaped structure (Fig 3G) having clear closed vascular bundle, which is an identifying characteristic feature of somatic embryogenesis. At the mature cotyledonary stage (Fig 3H) the photomicrograph from SEM clearly indicated two cotyledons along with shoot tip.

### Flow cytometry and ISSR analysis

**Fig 4:**
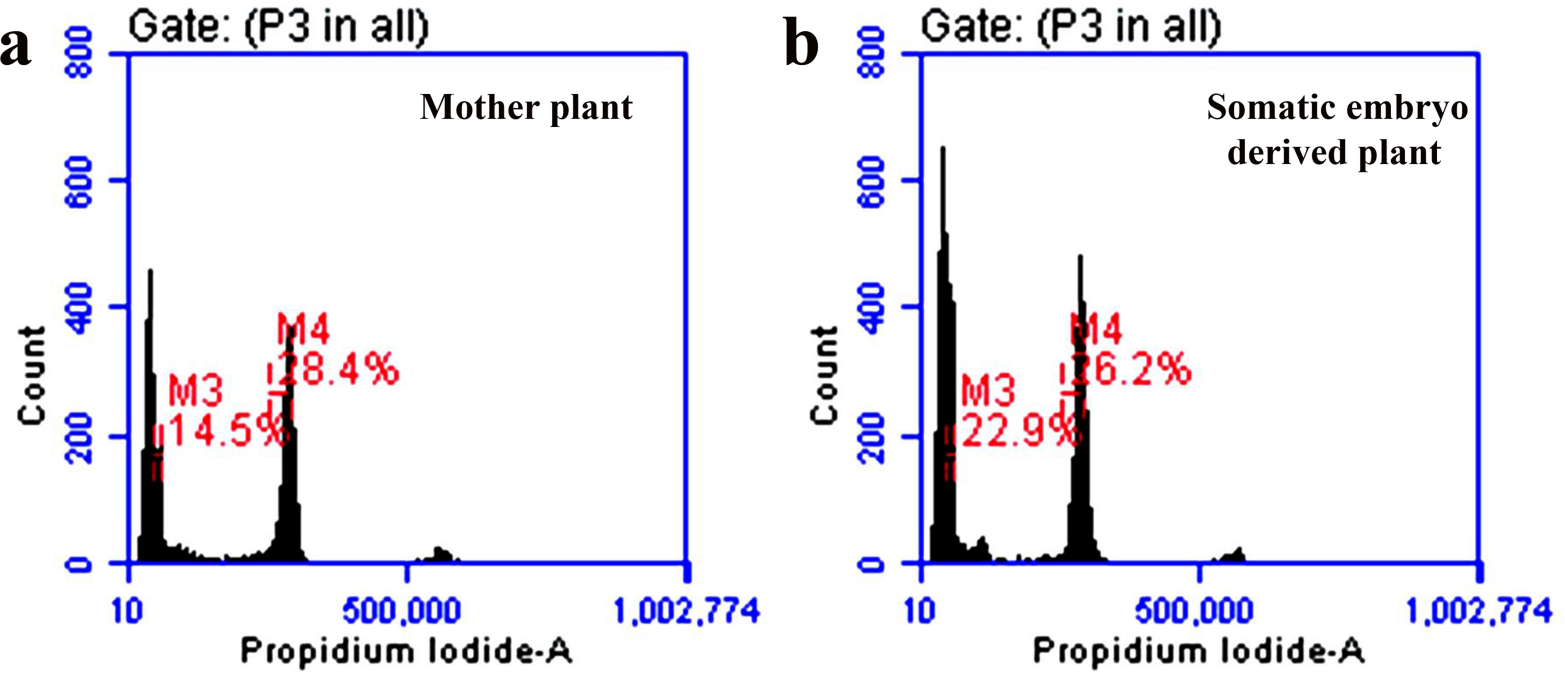
Flow cytometry profiles of the mother and somatic embryo derived plants of *H*. *sabdariffa* var. HS 4288. Histogram fluorescence intensity documenting the relative DNA content of the nuclear suspension from young roots of (a) mother (M4) and (b) somatic embryo derived plants (M4); in both figure a. and figure b., the peaks indicated as (M3) correspond to the internal reference standard of *Oryza sativa* (IR36).

Assessment of nuclear DNA stability and screening large numbers of somaclones within a short period of time by flow cytometry has become an indispensable tool in recent time [34], [39]. In flow cytometry analyses, the DNA content of the mother plant was compared with that of the somatic embryo derived plants by using *Oryza sativa* ‘IR36’ (1.01pg/2C) as a reference standard, where no statistically significant differences ((*p ≤ 0*.*05*) were obtained in the nuclear 2C DNA content between mother (5.90 ± 0.11 pg/2C) and somatic embryo (5.70 ± 0.02 pg/2C) derived plants (Table 4). The peaks in the histograms (Fig 4A and 4B) showed resemblance between these two types of plants.

**Table 4:** Nuclear 2C DNA content of the mother and somatic embryo derived plants of *H*. *sabdariffa* var. HS 4288.

Plant Material	DNA content (pg/2C ± SE)
Mother Plants	5.90±0.11
Somatic embryo derived plants	5.70±0.02

Values are expressed as mean ± standard error (±SE) of three independent experiments with ten plants from each group. The significance of difference between two means was compared by t–test at *p ≤0*.*05* level.

**Fig 5:**
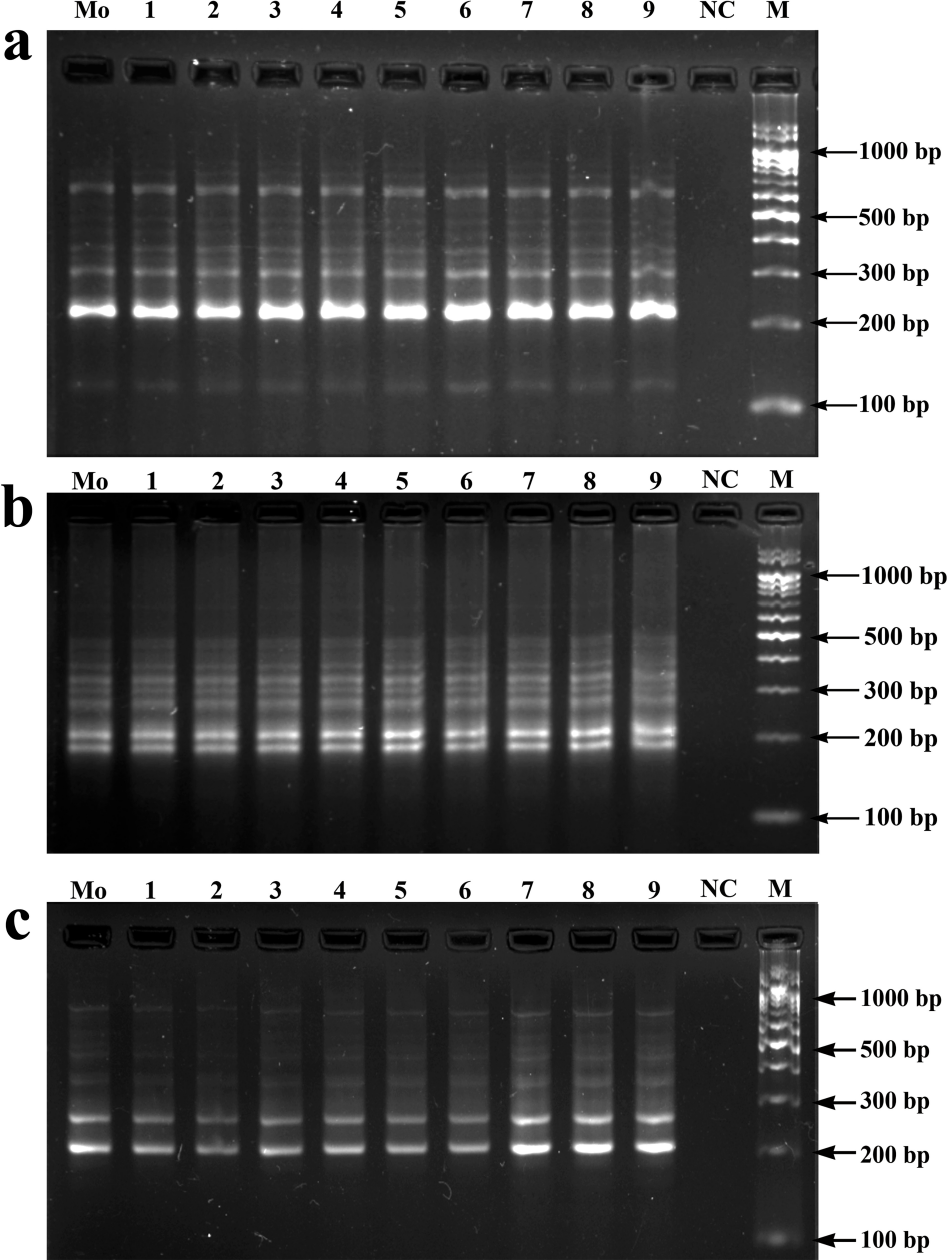
**Electrophoretic gel separation of the PCR amplified products by ISSR primers**; ISSR-4 (a), ISSR-8 (b) and ISSR-14 (c) (see Table 5 for primer details). Mo: mother plant (bulk); 1–9: somatic embryo derived plantlets; NC: negative control and M: 100 bp DNA ladder.

Besides flow cytometry, several DNA markers such as Random Amplification of Polymorphic DNA (RAPD), Amplified Fragment Length Polymorphism (AFLP), Restriction Fragment Length Polymorphism (RFLP) and microsatellite-based ISSR are frequently used to evaluate somaclonal variation within the regenerated plants [40], [28], [41]. Of these, ISSR marker analysis has been used most frequently because of its simplicity as well as high degree of sensitivity, increased stringency, higher reproducibility and cost-effectiveness [42]. In the present work with ISSR analysis, 15 ISSR primers (Table 5) resulted in 3–11 scorable bands ranging from 100 to 1000 bp. The frequencies of amplified loci obtained varied from primer to primer, such as among the di, tri and tetra nucleotide SSR motifs (AG, GA, AC, GT, CA, CT, TG, TC, CTC and GACA). Two motifs (AG and GA) produced maximum scorable loci, that reveal more coverage of the genome. The above observations were in conformity with the previous studies on *Swertia chirayita* [43] and in *Cymbopogon winterianus* [44]. Our results also suggested that primers anchored at 3' end developed higher resolution in banding profiles than primers anchored at 5' end, which is in accordance with the previous works by Blair et al. [45] with *Oryza sativa*. All the amplicons generated from the somatic embryo derived plantlets were monomorphic in nature and similar to those of the mother plant (Fig 5). The observations from both flow cytometry and ISSR analysis confirmed the genetic integrity of the regenerated plants developed through somatic embryogenesis.

**Table 5:** List of ISSR primers used for detecting the genetic stability in somatic embryo derived plants of *H*. *sabdariffa* var. HS 4288.

Sl. No.	Primer Code	Primer Sequence (5'–3')	Number of amplicon	Amplicon size range (bp)
nn1	ISSR-1	ACACACACACACACACTG	7	150–700
2	ISSR-2	TGTGTGTGTGTGTGTGCA	5	200–950
3	ISSR-3	GACAGACAGACAGACA	8	100–1000
4	ISSR-4	AGAGAGAGAGAGAGAGT	11	220–950
5	ISSR-5	CTCCTCCTCCTCGA	9	100–450
6	ISSR-6	CTCTCTCTCTCTAC	4	150–900
7	ISSR-7	TGTGTGTGTGTGTGTGGA	3	300–750
8	ISSR-8	GAGAGAGAGAGAGAGAC	10	180–750
9	ISSR-9	TCTCTCTCTCTCTCTCA	8	250–850
10	ISSR-10	AGAGAGAGAGAGAGAGTA	10	100–500
11	ISSR-11	ACACACACACACACAC	7	200–700
12	ISSR-12	GTGTGTGTGTGTGTGTA	5	350–900
13	ISSR-13	BDBCACACACACACACA	8	300–850
14	ISSR-14	DVDTCTCTCTCTCTCTC	8	200–800
15	ISSR-15	HBHAGAGAGAGAGAGAG	6	200–700

B = (T, C, or G); D = (A, T, or G,); V = (A, C, or G); H = (A, C, or T)

## Conclusions

A reproducible protocol for somatic embryogenesis from root derived calli of *Hibiscus sabdariffa* L. var. HS 4288 has been developed. The induction, maturation and regeneration of plantlets from somatic embryos have been clearly reported. Of the two culture media tested, DKW medium was superior to that of MS medium, and two PGRs (2, 4-D and KIN) have been found to be necessary for both somatic embryo induction and maturation, and BA with KIN for conversion into plantlets. Acclimatization of somatic embryo derived plantlets for growth in natural condition was also established for the first time. Genetic fidelity of the regenerants has been confirmed by flow cytometry and ISSR marker analyses. The development of *Agrobacterium rhizogenes* (Ri plasmid) induced transgenic root culture with novel gene(s) as mother root followed by induction of somatic embryogenesis and micropropagation of novel plantlets of this industrially important plant by the use of our protocol are distinct possibilities.

## Abbreviations

PGRs: Plant growth regulators
MS: Murashige and Skoog (1962)
DKW: Driver and Kuniyuki (1984)
2,4-D: 2,4 dichlorophenoxyacetic acid
KIN: Kinetin
BA: 6-Bezyladenine
NAA: α- Naphthalene acetic acid
PI: Propidium iodide
CV: Coefficient of variation
MFI: Median fluorescence intensity
PCA: Principal component analysis
SEM: Scanning electron microscopy
ISSR: Inter simple sequence repeat
